# Comparative Transcriptomic Analysis of the Liver and Spleen in Ussuri Catfish (*Pseudobagrus ussuriensis*) Challenged with Polyriboinosinic Polyribocytidylic Acid (Poly(I:C))

**DOI:** 10.3390/ani15162454

**Published:** 2025-08-21

**Authors:** Yu Liu, Ke Wang, Lingyun Lu, Huanhuan Miao, Libo Gu, Zhipeng Dou, Qing Liu

**Affiliations:** 1 College of Animal Science, Shanxi Agricultural University, Jinzhong 030800, China; 2 Shanxi Key Laboratory of Animal Genetics Resource Utilization and Breeding, Jinzhong 030800, China

**Keywords:** poly (I:C), key signaling pathways, core genes, Ussuri catfish

## Abstract

Poly (I:C), a viral mimic, is capable of activating the antiviral immune mechanisms in teleosts. In this study, we investigated the transcriptional responses of Ussuri Catfish (*Pseudobagrus ussuriensis*) to poly (I:C) stimulation at 3 and 48 h, focusing on the similarities and differences in antiviral mechanisms exhibited in the liver and spleen. At 3 h, the signaling pathways that were concurrently enriched in both the spleen and liver include JAK-STAT, TNF, NF-κB, RIG-I-like receptor, and NOD-like receptor. At 48 h, the signaling pathways that were concurrently enriched in both the spleen and liver include JAK-STAT signaling and cellular homeostasis processes. However, in the liver, the signaling pathways that responded to poly (I:C) stimulation at both 3 and 48 h are cytokine–cytokine receptor interaction and RIG-I-like receptor signaling. In the spleen, the signaling pathways that responded to poly (I:C) stimulation at both 3 and 48 h are Hippo signaling, Wnt signaling, TGF-β signaling, and ECM-receptor interaction. Ultimately, the pathways that were enriched in the intersection genes across all groups are JAK-STAT signaling, NK cell-mediated cytotoxicity, and ECM-receptor interaction, and the core genes identified in the intersection genes of all groups are *PTPRS*, *HECW1*, and *ERN1* (*IRE1*), along with *UMAD*, *DKK1*, *CSH*, and *RTKN2*. Through this study, we identified the key signaling pathways and core genes involved in the antiviral response of Ussuri catfish. These findings provide valuable insights into the antiviral mechanisms of Ussuri catfish.

## Simple Summary

This study looked at how Ussuri catfish defend against viruses. Scientists used a substance called poly (I:C) to mimic a viral infection and studied the fish’s liver and spleen after 3 and 48 h. Both organs activated similar antiviral pathways early on (like JAK-STAT and NF-κB), but later responses differed. The liver focused on immune signaling and virus detection, while the spleen used pathways linked to cell growth and tissue repair. Key genes (PTPRS, HECW1, ERN1, UMAD, DKK1, CSH, and RTKN2) were found across both organs and time points. These results reveal how Ussuri catfish fight viruses and highlight important genes for future research.

## 1. Introduction

As ectothermic vertebrates occupying a crucial evolutionary position, fish depend predominantly on their innate immune defenses to combat viral pathogens, with the efficacy of these antiviral mechanisms directly determining both ecological fitness and commercial viability in aquaculture systems [1]. At the molecular level, piscine immunity employs an array of pattern recognition receptors (PRRs), including Toll-like receptors (TLRs) and RIG-I-like receptors (RLRs), to identify pathogen-associated molecular patterns (PAMPs). This recognition initiates a cascade of antiviral defenses, most notably through the induction of type I interferon (IFN) pathways and the subsequent production of interferon-stimulated genes (ISGs), which collectively establish an antiviral state to limit viral propagation [2,3,4,5]. The synthetic dsRNA analog polyinosinic–polycytidylic acid [poly(I:C)] has emerged as a powerful tool for antiviral immunity research [6]. These attributes establish poly(I:C) as an indispensable experimental surrogate for dissecting the complexities of piscine antiviral immunity.

Exposure to the synthetic dsRNA analogue poly(I:C) robustly initiates antiviral immunity in teleost fish by sequentially engaging pattern-recognition receptors (PRRs) and downstream signaling cascades. In Qihe crucian carp (*Carassius auratus*), poly(I:C) up-regulated the Toll-like receptors CaTLR5 and CaTLR22 in multiple tissues, indicating their involvement in sensing pathogen-associated molecular patterns [7]. Similarly, the stimulation of peripheral blood leukocytes (PBLs) and head kidney leukocytes (HKLs) from common carp (*Cyprinus carpio* L.) with poly(I:C) markedly elevated the expression of the NOD-like receptor CcNLRC, whereas mandarinfish (*Siniperca chuatsi*) exhibited increased transcript levels of the RIG-I-like receptors MDA5 and LGP2 in spleen, gill, and head kidney after poly(I:C) or lipopolysaccharide challenge [8]. These PRR engagements converge on conserved signaling pathways. In Qihe crucian carp, poly(I:C) led to broad up-regulation of CaMyD88 and CaTRAF6, core adaptors of the TLR cascade [9]. In largemouth bass (*Micropterus salmoides*), transcriptomic profiling revealed robust activation of both NLR- and RLR-mediated signaling networks [10], while in yellow catfish (*Pelteobagrus fulvidraco*), poly(I:C) triggered the interferon regulatory factor (IRF)-dependent axis, including marked induction of IRF7 and IRF4 [11,12]. Collectively, these findings demonstrate that poly(I:C) serves as a potent activator of the teleost antiviral immune system by sequentially engaging TLRs, NLRs, RLRs, and their downstream adaptors and transcription factors.

The liver, a vital metabolic and immune organ in vertebrates, is particularly susceptible to viral attacks [13]. In fish, viral infections often lead to hepatic damage, disrupting metabolic homeostasis and impairing immune functions [14]. For instance, studies in zebrafish and grass carp have demonstrated that viral pathogens induce extensive inflammatory responses and apoptosis in liver tissues [15,16]. Similarly, the spleen, as a major lymphoid organ in fish, is a key target for viral replication and immune defense [17,18]. Viral infections can trigger significant histopathological changes in the spleen, including lymphocyte depletion and hematopoietic necrosis, ultimately compromising adaptive immunity [19,20]. Transcriptomic analyses in other fish species have revealed that viral challenges alter the expression of immune-related genes in the spleen, highlighting its central role in antiviral defense [21].

In recent years, the Ussuri catfish (*Pseudobagrus ussuriensis*), a premium aquaculture species, has experienced rapid expansion in farming scale. As a representative member of the family Bagridae, elucidating its antiviral immune mechanisms not only provides a theoretical basis for the selective breeding of disease-resistant lines, but also serves as a valuable reference for deciphering the broader immune architecture of bagrid fishes. However, viral diseases remain a major bottleneck, affecting production efficiency and profitability in Ussuri catfish aquaculture [22,23,24,25]. Through antiviral research, targeted prevention and control measures can be developed to reduce the incidence and spread of viral diseases [26], minimize economic losses caused by such diseases, enhance the sustainability of Ussuri catfish aquaculture, and safeguard the healthy development of the fisheries industry. Moreover, the antiviral research on Ussuri catfish may provide valuable references for antiviral studies in other fish species and even broader aquatic organisms, driving advancements in the entire field of aquatic antiviral research and offering profound scientific value and broad application prospects.

## 2. Materials and Methods

### 2.1. Ethics Statement

This study was conducted in accordance with the National Research Council’s Guide for the Care and Use of Laboratory Animals and approved by the Animal Ethical Committee of Shanxi Agricultural University (Shanxi, China) (Approval No.: SXAU-EAW-2025Pu.ZZ.001018260). Fish were treated with ethyl-3-aminobenzoate methanesulfonate (MS-222) (Sigma, Co.St, Louis, MO, USA) in order to harvest tissue.

### 2.2. Fish Handling and Trial Design

The juvenile *P. ussuriensis* utilized in this study were self-propagated in our lab. Parents from Shanxi, China’s Hutuo River made up the breeding stock. A carefully chosen group of healthy, disease-free animals (mean weight of 7.56 ± 1.02 g and mean length of 7.57 ± 0.44 cm) were acclimated for two weeks in a 100 L aquarium with recirculating filtration systems, keeping the temperature at 15–20 °C and the dissolved oxygen levels above 5 mg/L [22,27,28]. Eliminating any stressors associated with environmental changes and transportation was the goal of this acclimatization phase. Individuals of *P. ussuriensis* were given commercial yellow catfish (*Tachysurus fulvidraco*) pellet feed (made by Tongwei Co. Ltd., Chengdu, China) twice a day during the temporary feeding period.

Sixty healthy individuals with a similar size (average weight: 7.98 ± 1.05 g) were randomly divided into two groups, at 30 fish per group. Each fish of the control group was intraperitoneally injected with 100 µL phosphate buffered saline (PBS) reagent, while the treatment group was injected with 100 µL of 1 mg/mL poly (I:C). The sampling time was in accordance with previous research [29,30]. Briefly, each group contained three biological replicates, with a total of 9 fish (3 fish as a sample, *n* = 3), and the fish were dissected to collect the liver and spleen samples at 3 h and 48 h post-injection. Specifically, the 3 h samples consisted of PBS-3L (PBS-treated liver), PBS-3S (PBS-treated spleen), IC-3L (Poly(I:C)-treated liver), and IC-3S (Poly(I:C)-treated spleen), while the 48 h samples followed the same nomenclature (PBS-48L, PBS-48S, IC-48L, and IC-48S). The liver or spleen from each fish was quickly put into a single frozen pipe, snap-frozen in the liquid nitrogen, and finally transported to store at −80 °C until use.

### 2.3. RNA Extraction, Library Construction, Sequencing

The liver and spleen RNA of each fish were separately extracted, and the RNA of each biological replicate was composed of an equal mass mixing of the RNA from the three fish. All RNA extractions were performed using TRlzol reagent (Invitrogen, Carlsbad, CA, USA), and then the integrity and purity were identified via 1% (*w*/*v*) agarose gel, a Nanodrop 1000 spectrophotometer (ThermoFisher Scientific, Waltham, MA, USA), and Agilent 2100 (Agilent, Agilent Technologies, Inc., Carlsbad, CA, USA). RNA samples with high quality (A260/A280 > 1.8; A260/A230 > 1.8) were picked for the library construction, high-throughput sequencing, and subsequent quantitative real-time PCR experiments.

After enrichment with oligo-dT beads, the purified mRNA of each sample was treated with the NEBNext ^®^ UltraTM RNA Library Prep Kit for Illumina ^®^ (NEB, Ipswich, MA, USA) according to the manufacturer’s instructions, and we subsequently completed the first-strand-cDNA and second-strand-cDNA synthesis. The double-stranded cDNA was purified using QIAquick PCR Purification columns (Qiagen, Valencia, CA, USA), end repaired, and added to the poly A tail; then, the sequencing joint was attached. Finally, the product was purified again, and the library quality was evaluated by an Agilent 2100 system; then, if applicable, the 24 cDNA libraries were sequenced on an Illumina HiSeq 2000. The paired-end read was 125 bp/150 bp (Novogene, Beijing, China).

### 2.4. Quality Control and De Novo Assembly

For the entire reference-guided transcriptome project, comprehensive quality control was performed with RSeQC to evaluate (i) sequencing saturation, (ii) randomness of library construction, and (iii) read distribution across genomic features. High-quality clean reads were then aligned to the designated reference genome using HISAT2 [25], yielding positional information on both the genome and annotated genes, together with sample-specific sequence signatures. Leveraging the reference gene sequences and their functional annotations as a database, protein-coding transcripts were identified and quantified by sequence-similarity alignment. Finally, read counts per gene were obtained with HTSeq-count, providing accurate expression abundance estimates for each sample.

### 2.5. Functional Annotation

The function of the unigenes was annotated by alignment of the unigenes with the NCBI nonredundant (NR), SwissProt, and Clusters of orthologous groups for eukaryotic complete genomes (KOG) databases using Blastx, with a threshold E-value of 10^−5^. Proteins with the highest hits to the unigenes were used to assign functional annotations. Based on the SwissProt annotation (https://ngdc.cncb.ac.cn/databasecommons/database/id/5614) (accessed on 1 January 1999), Gene Ontology (GO) classification (http://geneontology.org/) (accessed on 1 March 2011) was performed using the mapping relation between SwissProt and GO terms. Unigenes were mapped to the Kyoto Encyclopedia of Genes and Genomes (KEGG) database (http://www.genome.jp/kegg/) (accessed on 1 May 1995) to annotate potential metabolic pathways.

### 2.6. Analysis of Differentially Expressed Unigenes (DEGs), Cluster Analysis, and GO and KEGG Enrichment

FPKM and read count values of each gene were calculated using bowtie2 and eXpress. DEGs were identified using the DESeq (2012) function estimateSizeFactors and the nbinom test. A *p*-value of 0.05 and a fold change > 2 were set as the threshold for significantly differential expression. Hierarchical cluster analysis of the DEGs was performed to explore transcript expression patterns. GO enrichment and KEGG pathway enrichment analyses of DEGs were performed using R software (v 4.3.0) based on a hypergeometric distribution. Transcriptome data analysis software is listed in Table 1.

### 2.7. Quantitative Real-Time PCR (qPCR)

Quantitative real-time PCR (qPCR) was performed to verify the hub genes and the RNA-seq results. qPCR primers (Table 2) were designed based on annotated gene sequences from the transcriptome data sequence. The first-strand cDNA was synthesized from 1 μg total RNA using a PrimeScript RT reagent kit (Takara, Tokyo, Japan). *β-actin* was used as an internal reference gene to normalize the gene expression level [31]. qPCR was performed using SYBR Green Premix Ex Taq (Takara) on a CFX96 Real-Time PCR System (Bio-Rad, Hercules, CA, USA). The program for qRT-PCR was 95 °C for 30 s and 40 cycles of amplification at 95 °C for 5 s, 59.5 °C for 20 s, and 72 °C for 15 s. A standard curve was generated to assess accuracy, and primers with an amplification efficiency of more than 95% were selected for qPCR. Four biological and three technical replicates were used for each gene. The relative expression level of genes was analyzed using the 2^−△△CT^ method [32].

### 2.8. Statistical Analysis

Statistical analyses were performed using GraphPad Prism 8. Values are expressed as mean ± SEM.

## 3. Results

### 3.1. Overview of RNA-Seq Data

In this experiment, transcriptome sequencing was conducted on 24 samples, resulting in a total of 146.64 G of clean data. The effective data volume for each sample ranged from 6.08 G to 7.34 G. The Q30 base distribution ranged from 94.42% to 96.93%, with an average GC content of 43.98%. Detailed information is provided in Table 3.

### 3.2. Transcriptomic Responses in Liver and Spleen of P. ussuriensis at 3 h Post-Poly(I:C) Stimulation

**Figure 1:**
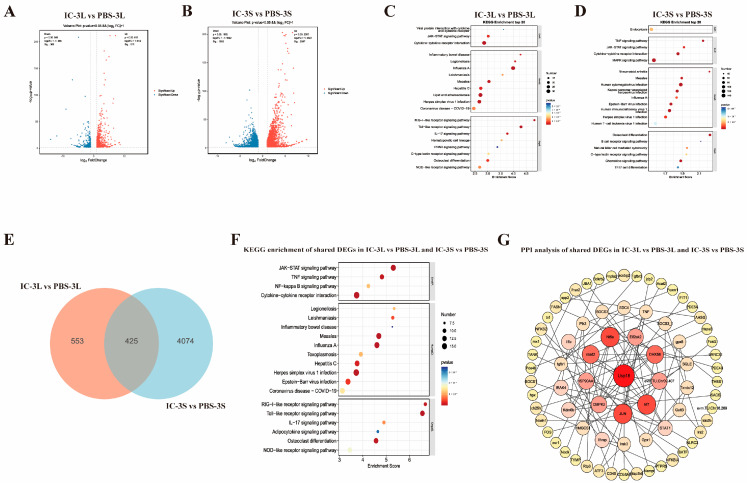
Transcriptomic analysis of liver and spleen in *P. ussuriensis* at 3 h post-Poly(I:C) stimulation. (**A**,**B**) Volcano plots showing differentially expressed genes (DEGs) in liver (IC-3L vs. PBS-3L) (**A**) and spleen (IC-3S vs. PBS-3S) (**B**); (**C**,**D**) KEGG pathway enrichment of DEGs in liver (IC-3L vs. PBS-3L) (**C**) and spleen (IC-3S vs. PBS-3S) (**D**); (**E**) Venn diagram between liver and spleen at 48 h post-Poly(I:C) stimulation; (**F**) KEGG analysis of shared DEGs; (**G**) PPI network of shared DEGs.

To elucidate the early immune responses to viral mimicry in *P. ussuriensis*, we conducted transcriptome sequencing of liver and spleen tissues at 3 h post-Poly(I:C) challenge. DEG analysis revealed substantial transcriptomic reprogramming in both liver (IC-3L vs. PBS-3L) and spleen (IC-3S vs. PBS-3S). The lists of differentially expressed genes for the comparisons IC-3L vs. PBS-3L (liver) and IC-3S vs. PBS-3S (spleen) are provided in Supplementary Material S1 and S2, respectively. As illustrated in the volcano plots (Figure 1A,B), a large number of genes were significantly upregulated or downregulated upon Poly(I:C) stimulation. Specifically, 978 DEGs were identified in the liver (612 upregulated, 366 downregulated), whereas the spleen exhibited a more pronounced response, with 4499 DEGs (2597 upregulated, 1902 downregulated). Notably, KEGG pathway enrichment analysis (Figure 1C,D) demonstrated significant enrichment of multiple immune-related pathways in both tissues. In the liver, key pathways included the JAK-STAT signaling pathway, TNF signaling pathway, NF-κB signaling pathway, RIG-I-like receptor signaling pathway, and cytokine–cytokine receptor interaction. In contrast, the spleen exhibited additional enriched pathways, such as Toll-like receptor signaling, IL-17 signaling, and T-cell differentiation, highlighting its specialized function in adaptive immunity. To uncover shared immune mechanisms between the two tissues, we performed an intersection analysis of liver- and spleen-specific DEGs, identifying 425 common DEGs (Figure 1E). KEGG enrichment analysis of these shared genes (Figure 1F) revealed their predominant involvement in JAK-STAT, TNF, NF-κB, RIG-I-like receptor, NOD-like receptor, and Toll-like receptor signaling pathways, along with multiple viral infection-related pathways (e.g., influenza A, measles, and herpes simplex virus infection). Furthermore, protein–protein interaction (PPI) network analysis of the 425 shared DEGs (Figure 1G) identified several potential core regulatory factors, including Usp18, Irf7, Ifit1, and Jun, which may play pivotal roles in orchestrating the antiviral immune response.

### 3.3. Transcriptomic Responses in Liver and Spleen of P. ussuriensis at 48 h Post-Poly(I:C) Stimulation

**Figure 2:**
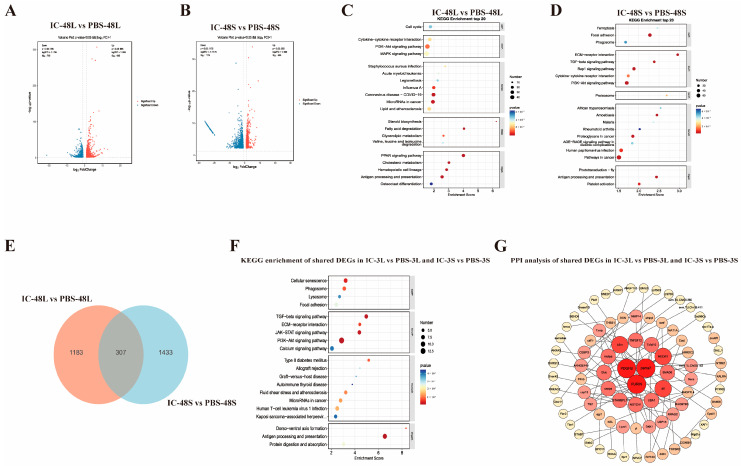
Transcriptomic response of liver and spleen in *P. ussuriensis* at 48 h post-Poly(I:C) stimulation. (**A**,**B**) Volcano plots showing DEGs in liver (IC-48L vs. PBS-48L) and spleen (IC-48S vs. PBS-48S). (**C**,**D**) KEGG pathway enrichment of DEGs in liver (IC-48L vs. PBS-48L) (**C**) and spleen (IC-48S vs. PBS-48S) (**D**). (**E**) Venn diagram between liver and spleen at 48 h post-Poly(I:C) stimulation. (**F**) KEGG pathways of shared DEGs. (**G**) PPI network of shared DEGs.

To characterize the transcriptional landscape underlying the late-phase immune response, we compared hepatic (IC-48L vs. PBS-48L) and splenic (IC-48S vs. PBS-48S) transcriptomes of *P. ussuriensis* 48 h after Poly(I:C) stimulation. Differential-expression analysis (Figure 2A,B) revealed robust transcriptional reprogramming in both tissues. In liver, 1490 genes were significantly altered, with 695 up-regulated and 795 down-regulated. In spleen, the response was even more extensive, with 1740 differentially expressed genes—565 up-regulated and 1175 down-regulated. The lists of differentially expressed genes for the comparisons IC-48L vs. PBS-48L (liver) and IC-48S vs. PBS-48S (spleen) are provided in Supplementary Material S3 and S4, respectively.

Transcriptomic analysis of *P. ussuriensis* at 48 h post-Poly(I:C) stimulation revealed distinct tissue-specific responses, with the liver showing 1490 differentially expressed genes primarily involved in metabolic regulation through PI3K-Akt and MAPK signaling pathways, while the spleen exhibited 1740 differentially expressed genes predominantly associated with immune function and tissue remodeling, including TGF-β signaling and antigen processing pathways (Figure 2C,D). Intersection analysis identified 307 conserved differentially expressed genes that form an integrated regulatory network (Figure 2E–G), with key hub genes (FURIN, TGFB1, MMP9) coordinating shared pathways, such as JAK-STAT signaling and cellular homeostasis processes.

### 3.4. Temporal Analysis of Shared Immune-Related Genes in Liver Following Poly(I:C) Stimulation

**Figure 3:**
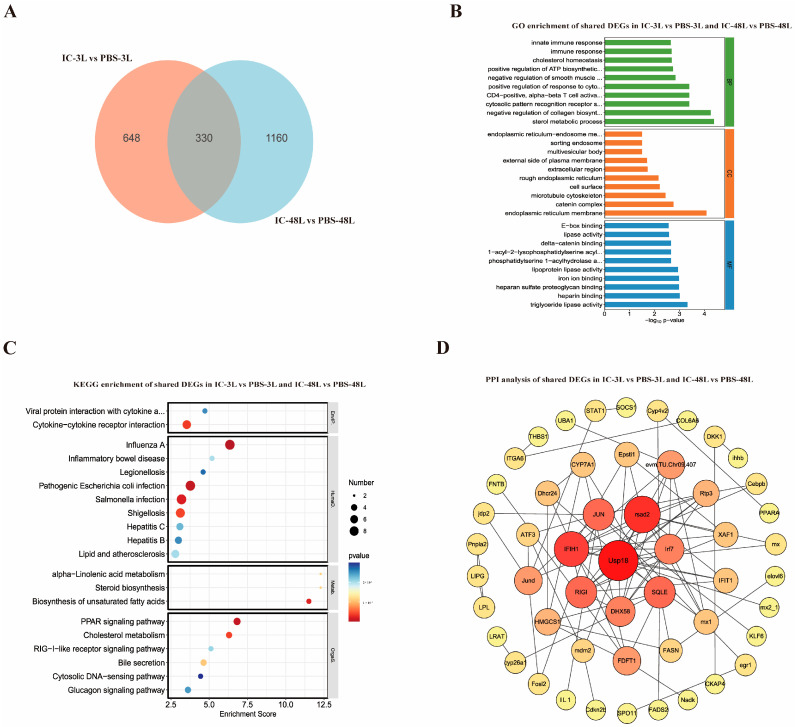
Overlapping transcriptomic response in liver of *P. ussuriensis* at 3 h and 48 h post-Poly(I:C) stimulation. (**A**) Venn diagram between IC-3L and IC-48L groups; (**B**) GO enrichment analysis of shared DEGs; (**C**) KEGG analysis of shared DEGs; (**D**) PPI network of shared DEGs.

To elucidate the conserved transcriptional response in the liver during Poly(I:C)-induced immune activation, we identified 330 shared DEGs between early (3 h) and late (48 h) time points through intersection analysis (Figure 3A). GO enrichment analysis (Figure 3B) revealed these genes were significantly associated with innate immune response, lipid metabolic regulation, and antiviral defense in biological processes; were localized to plasma membrane, endoplasmic reticulum, and lipid droplets in cellular components; and exhibited molecular functions, including lipase activity and protein binding. KEGG pathway analysis (Figure 3C) further demonstrated their involvement in critical immune pathways (cytokine–cytokine receptor interaction, RIG-I-like receptor signaling) and metabolic processes (cholesterol metabolism, fatty acid oxidation). Protein–protein interaction network analysis (Figure 3D) identified several hub genes (Usp18, Stat2, Ifih1, Jun, Irf7, Rsad2, Ifit1, Mx1) that likely play central roles in coordinating the antiviral immune response and immunometabolic regulation across different phases of infection.

### 3.5. Temporal Analysis of Shared Immune-Related Genes in Spleen Following Poly(I:C) Stimulation

**Figure 4:**
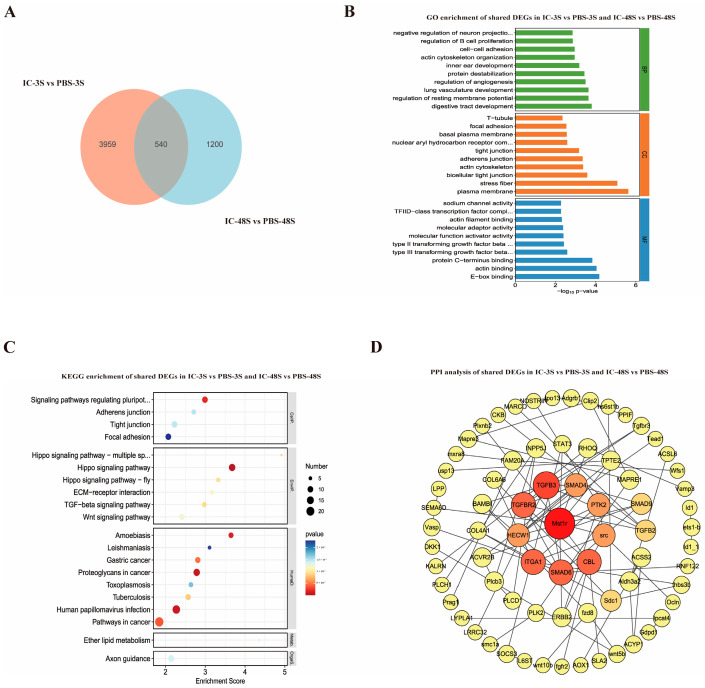
Overlapping transcriptomic response in spleen of *P. ussuriensis* at 3 h and 48 h post-Poly(I:C) stimulation. (**A**) Venn diagram between IC-3S and IC-48S; (**B**) GO enrichment analysis of shared DEGs; (**C**) KEGG enrichment analysis of shared DEGs; (**D**) PPI network analysis of shared DEGs.

To elucidate the sustained immune response mechanisms in the spleen from early to intermediate phases following Poly(I:C) stimulation, we conducted an intersection analysis of DEGs between IC-3S vs. PBS-3S and IC-48S vs. PBS-48S. Venn diagram analysis (Figure 4A) identified 540 consistently differentially expressed genes across both time points. GO functional annotation of these shared DEGs (Figure 4B) revealed significant enrichment in biological processes (BPs), including regulation of B cell proliferation, negative regulation of neuron projection, endothelial cell–cell junction formation, angiogenesis regulation, and ion channel activity modulation, suggesting their involvement in immune cell function, tissue homeostasis maintenance, and inflammatory microenvironment regulation. Cellular component (CC) analysis showed predominant localization to tight junctions, T-tubules, the external side of the plasma membrane, and the basement membrane, indicating these gene products likely function at cell–cell communication interfaces and participate in immune cell migration and tissue architecture modulation. Molecular function (MF) analysis demonstrated enrichment in transcription factor binding, calcium ion binding, protease activity regulation, and E-box sequence recognition, highlighting their regulatory potential in immune-related gene expression. KEGG pathway analysis (Figure 4C) of the shared DEGs identified significant enrichment in Hippo signaling, Wnt signaling, TGF-β signaling, ECM–receptor interaction, and cell adhesion-related pathways (adherens junction, tight junction, focal adhesion), along with cancer-related pathways (gastric cancer) and protozoan infection pathways (Leishmania infection, toxoplasmosis). Protein–protein interaction network analysis (Figure 4D) of these 540 shared DEGs revealed critical regulatory hubs, including (1) TGFB1, TGFBR1, TGFBR2, SMAD2, and SMAD3 as core components of TGF-β signaling; (2) ITGA4, ITGA1, ITGA5, PTK2, SRC, CBL, and MET, involved in cell adhesion, signal transduction, and migration regulation; and (3) RHOG, NPR3, OLR1, and HECW1, participating in oxidative stress response, metabolic regulation, and cellular communication, collectively forming an integrated network governing the spleen’s sustained immune response to viral challenge.

### 3.6. Analysis of Shared DEGs Among All Comparison Groups

**Figure 5:**
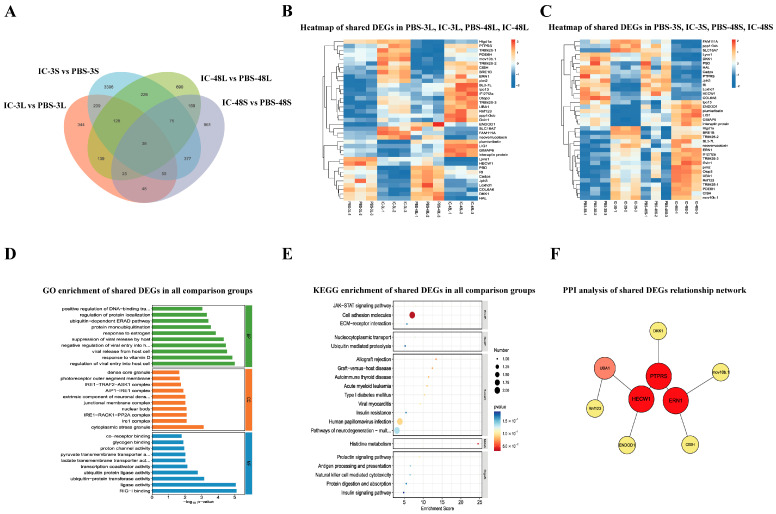
Conserved transcriptomic response across all timepoints and tissues in *P. ussuriensis* following Poly(I:C) stimulation. (**A**) Venn diagram across four comparisons: IC-3L, IC-3S, IC-48L, and IC-48S; (**B**,**C**) heatmaps of shared DEGs showing consistent expression patterns in liver (**B**) and spleen (**C**) across timepoints; (**D**) GO enrichment of shared DEGs; (**E**) KEGG enrichment of shared DEGs; (**F**) PPI network of shared DEGs.

To elucidate the core immune response mechanisms universally activated across tissues and time points in *P. ussuriensis* following Poly(I:C) stimulation, we performed a comprehensive intersection analysis of DEGs from liver and spleen tissues at both 3 h and 48 h post-treatment. Venn diagram analysis (Figure 5A) identified 39 conserved DEGs that were consistently regulated across all four comparison groups (IC-3L, IC-3S, IC-48L, IC-48S). Heatmap visualization (Figure 5B,C) revealed distinct expression dynamics: hepatic genes (Figure 5B) exhibited sustained upregulation with strong temporal persistence, while splenic genes (Figure 5C) displayed tissue-specific patterns with early (3 h) peak expression followed by moderate attenuation, suggesting their involvement in rapid initial responses. GO enrichment analysis (Figure 5D) demonstrated these core genes were significantly associated with (1) biological processes including interferon-mediated regulation, viral replication suppression, protein localization control, and post-translational modifications (e.g., ubiquitination); and (2) molecular functions such as ubiquitin-protein ligase activity, transporter binding, transcription factor interaction, and RIG-I binding capacity. KEGG pathway analysis (Figure 5E) further highlighted their involvement in JAK-STAT signaling, antigen processing/presentation, NK cell-mediated cytotoxicity, ECM–receptor interaction, ubiquitin-mediated proteolysis, and immunometabolic crosstalk pathways (viral myocarditis, diabetes, graft-versus-host disease). Protein–protein interaction network analysis (Figure 5F) identified key regulatory hubs, including PTPRS, HECW1, and ERN1 (IRE1), along with UMAD, DKK1, CSH, and RTKN2, forming an integrated network orchestrating the organism-wide antiviral defense.

### 3.7. Validation of Key Genes by qPCR

**Figure 6:**
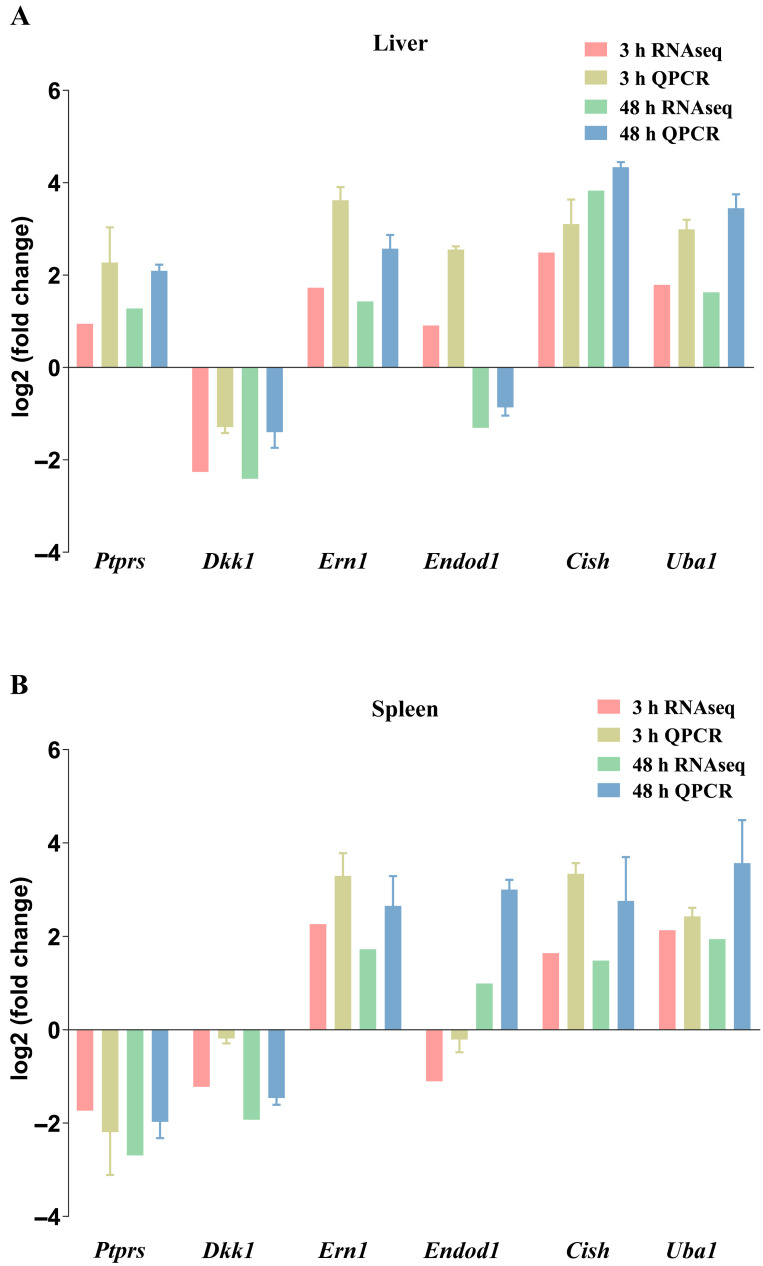
Comparison of gene expression data between RNA-Seq and Q-PCR in liver (**A**) and spleen (**B**).

To further validate the reliability of the transcriptomic analysis results, we performed qRT-PCR on six randomly selected DEGs from four comparisons: IC-3L, IC-3S, IC-48L, and IC-48S groups. The specific genes selected for validation are listed in Table 2. The results showed that the expression levels of the genes detected by qRT-PCR exhibited a similar trend to the RNA-Seq results (Figure 6A,B). This consistency between qRT-PCR and RNA-Seq data confirms the reliability of our RNA-Seq findings.

## 4. Discussion

The present study provides a comprehensive characterization of the spatiotemporal immune responses in *P. ussuriensis* following Poly(I:C) challenge, revealing conserved yet tissue-specific antiviral mechanisms in teleost fishes. Our findings demonstrate that both hepatic and splenic tissues employ a biphasic strategy, transitioning from acute immune activation to homeostasis restoration, while maintaining distinct functional specializations that collectively constitute an integrated host defense system.

### 4.1. Molecular Mechanisms of Innate Immune Responses and Maintenance of Tissue Homeostasis in the Liver

In the liver, we observed a remarkable temporal shift from early-phase interferon-dominated responses (3 h) to late-phase metabolic reprogramming (48 h). The initial antiviral state was characterized by robust activation of JAK-STAT and RLR pathways, consistent with previous reports in *Ctenopharyngodon idella* [33,34,35]. Particularly noteworthy was the central role of IFIH1-mediated dsRNA sensing, which initiates IRF3/7-dependent interferon production a conserved mechanism across aquatic vertebrates [36,37]. The subsequent metabolic transition, featuring PPAR and PI3K-Akt pathway activation, reflects the liver’s unique capacity to couple immune defense with energy homeostasis, as documented in *Micropterus salmoides* [38,39,40,41]. In the PPI network constructed from the differentially expressed genes shared between 3 h and 48 h in the liver, a tightly interconnected core structure centered on the interferon signaling pathway was formed. The central nodes, including Usp18, IFIH1, Rsad2, Irf7, JUN, and RIGI (DDX58), exhibited significantly high connectivity, indicating their pivotal regulatory roles in the Poly I:C-induced liver response process. Usp18, as a negative regulator of interferon signaling, clearly controls the intensity of IFN responses and prevents immune overactivation [42]. IFIH1 (MDA5) and RIGI (DDX58), as key pattern recognition receptors for detecting dsRNA, are responsible for sensing Poly I:C and activating downstream IRF7, thereby driving interferon production [43]. Rsad2 (Viperin) and other classic ISGs, such as MX1 and IFIT1, are located at the periphery of the network and are directly associated with antiviral effects [44]. Notably, several nodes involved in lipid metabolism (e.g., FDFT1, SQLE, HMGCS1, and FASN) are also integrated into this immune interaction network, indicating the presence of an immune–metabolic crosstalk mechanism in the liver during the response process. Additionally, the transcription factor JUN, as a regulatory hub for inflammation and regeneration, connects multiple immune and metabolism-related nodes, highlighting its bridging role in transcriptional regulatory integration. Overall, this network is centered on Usp18 as a feedback regulatory hub, with the RLR-IFN axis as the main trunk and JUN as a cross-pathway connector, integrating lipid metabolism factors to form a multi-layered synergistic network. This supports the dynamic response chain in the liver following Poly I:C stimulation, from pathogen recognition to signal amplification, antiviral response, and negative feedback regulation. This network pattern reflects the liver’s systemic integration capability in innate immune responses and serves as a crucial foundation for maintaining tissue homeostasis and controlling immune intensity.

### 4.2. Multiple Functioning of the Spleen as a Basis for Structural Regulation and Immune Coordination

This study systematically analyzed the transcriptomic responses of the spleen of *P. ussuriensis* at two critical time points, 3 h and 48 h, following Poly I:C stimulation. It revealed a continuous dynamic process in the spleen shifting from immune activation to tissue homeostasis recovery during the simulated viral infection response, showcasing unique tissue-specific functions in structural regulation and immune coordination. In the early 3 h stage, the spleen rapidly activated multiple typical antiviral and inflammation-related signaling pathways. KEGG enrichment analysis indicated that pathways such as JAK-STAT, TNF, CCRI, MAPK, Th17 cell differentiation, NK cell-mediated cytotoxicity, BCR, and CLR were significantly enriched, characterized by low *p*-values and a high number of enriched genes. This suggests that the spleen undertakes multiple functions in the early stage, including pathogen recognition, cytokine release, and the activation of immune effector cells. Specifically, the JAK-STAT pathway mediates the expression of interferon-stimulated genes (ISGs) and is a crucial module for type I IFN signaling [45]. The TNF and MAPK pathways amplify the inflammatory cascade by inducing pro-inflammatory cytokines and chemokines, such as IL-1β and CXCLs [46,47]. Moreover, the enrichment of BCR and NK cell pathways indicates that both adaptive and innate immune cells are mobilized into an active state [48,49]. This broad-spectrum, rapid, and diverse cell type activation pattern has also been repeatedly validated in other fish species, such as channel catfish and yellow croaker, and is a common immune activation feature following Poly I:C stimulation.

As the stimulation extended to 48 h, the expression profile and pathway enrichment structure of the spleen underwent a significant shift, trending from inflammatory activation towards immune regulation and tissue structural remodeling. At this point, significantly enriched pathways included TGF-β, ECM-receptor interaction, PI3K-Akt, AGE-RAGE, proteasome, antigen processing and presentation, platelet activation, malaria, and cancer signaling pathways. The TGF-β signaling pathway, as a negative regulatory pathway, plays a central role in suppressing excessive immune responses and promoting tissue repair [50]. Its co-enrichment with ECM receptor pathways, adhesion junctions, and tight junctions indicates that the spleen begins to initiate structural recovery and microenvironmental homeostasis regulation at this stage. More importantly, the 540 differentially expressed genes (DEGs) shared between the two time points were enriched in pathways such as Hippo, Wnt, TGF-β, ECM-receptor interaction, and focal adhesion, forming the intersection core of the immune signaling and cellular structural signaling networks. In the PPI network constructed from the commonly differentially expressed genes in the spleen at 3 h and 48 h, Mst1r emerged as a highly connected central node, indicating its significant regulatory role in the sustained immune response induced by Poly I:C. Mst1r, together with TGFB3, TGFBR2, SMAD4, and SMAD6, constitutes the TGF-β signaling module, suggesting that this pathway may mediate immune suppression and tissue repair in the later stages of inflammation. Meanwhile, ITGA1, PTK2, and SRC form the cell adhesion and focal adhesion signaling axis, participating in immune cell migration, homing, and local structural remodeling [51,52,53]. This network integrates three types of functional pathways: signal regulation, adhesion movement, and immune homeostasis, revealing a multi-pathway structure centered on Mst1r, with TGF-β as the backbone and the integrin-adhesion module as the framework. This supports the spleen’s functional shift from immune activation to inflammation resolution and microenvironmental repair following Poly I:C stimulation.

### 4.3. Coherence and Appropriateness of the Immune Response as a Consequence of Co-Activation Key Pathways in the Liver and Spleen

After analyzing the differentially co-expressed genes in the liver and spleen under Poly I:C stimulation, KEGG pathway enrichment analysis further revealed that these shared genes are primarily concentrated in several classic antiviral and immune regulation pathways. These include the JAK-STAT signaling pathway, RIG-I-like receptor pathway, natural killer cell-mediated cytotoxicity, antigen processing and presentation, protein ubiquitination degradation, ECM–receptor interaction, and insulin signaling regulation, among others. These findings indicate that under Poly I:C stimulation, the liver and spleen not only share similar immune activation pathways but also achieve a closed-loop regulation of antiviral recognition, signal transmission, effector execution, and negative regulation through coordinated molecular functions. The constructed protein–protein interaction (PPI) network further revealed the regulatory structure formed by this core set of genes. In this network, PTPRS, ERN1, and HECW1 emerged as central nodes, forming a stable and tightly interacting signaling regulatory network. PTPRS, as a protein tyrosine phosphatase, likely modulates the threshold and duration of immune responses by regulating the JAK-STAT pathway [54]. ERN1 (IRE1), an endoplasmic reticulum stress sensor, is considered a key component in signal amplification during the viral infection response when activated in conjunction with the RIG-I complex [55]. HECW1, an E3 ubiquitin ligase, may regulate the degradation of key signaling proteins to either amplify or terminate signals [56]. The interactions among these proteins demonstrate that, based on consistent gene expression, different tissues also achieve high coordination at the protein level, ensuring the coherence and appropriateness of the immune response.

## 5. Conclusions

This study systematically analyzed the transcriptomic responses of the liver and spleen of *P. ussuriensis* under Poly (I:C) stimulation. It was found that both tissues exhibited a temporal shift from immune activation to homeostatic regulation, with each displaying unique functional characteristics. The liver was primarily involved in interferon-mediated antiviral pathways and metabolic restructuring, while the spleen focused more on immune signal integration and tissue repair. Both tissues were enriched in core pathways such as JAK-STAT and RLR, and they constructed a cross-tissue shared immune regulatory network through key nodes like Usp18, Irf7, Mst1r, and TGFB. Ultimately, a multi-stage coordinated response pattern covering “pathogen recognition—signal amplification—immune execution—negative regulation—structural recovery” was formed, providing important molecular evidence for a deeper understanding of the broad-spectrum immune regulatory mechanisms in fish.

## Figures and Tables

**Table 1:** Software suite for transcriptomic data analysis.

Software	Version	Parameters	Function
DESeq	1.34.1	qvalue(pvalue)<0.05, |log2FoldChange|>1	Non-biological repeated difference analysis
htseq-count	0.11.2	-s reverse	Gene quantification
hisat2	2.1.0	--rna-strandness rf --fr	genomic alignment
DESeq2	1.22.2	qvalue(pvalue)<0.05, |log2FoldChange|>1	Biological duplication and paired difference analysis
fastp	0.20.1	--length_required 50	Original read base quality control
fastqc	v0.11.9	default	Raw read quality assessment
RseQC	4.0.0	default	RNA quality control
samtools	1.9	mpileup -uRf -d 1000000	sam and bam file analysis

**Table 2:** Sequences of the primers used in this study.

Gene Name	Sequences (5′-3′)
*Ptprs*	F: TAAAGGGCTATCGGGTT
	R: GCTCTGGATGGTGGTGA
*Dkk1*	F: CTGCTTACTGTCCCGTG
	R: GTAGCGTTTGCCTGATG
*Ern1*	F: ACCGAGACATCAAACCG
	R: AACATTCCTGCCACCTG
*Endod1*	F: GGAGAGCGAGAGACCAG
	R: GGAGCACCAGAGAGAGG
*Cish*	F: CAGGACGAAGAGTGTGA
	R: CAAGATGCTGTAGGGAT
*Uba1*	F: CTGCTATCGCCACAACC
	R: GAATACTCTTTCCCCGC
*β-actin*	F: AGAGCGTAACCCTCGTAG R: CTGCTTTGCGGCTGAATA

**Table 3:** Results of the sequencing data quality.

Sample	RawReads (M)	RawBases (G)	CleanReads (M)	CleanBases (G)	ValidBases (%)	Q30 (%)	GC (%)
IC-3L-1	24.87	7.24	23.99	6.99	96.48	96.41	44.61
IC-3L-2	24.76	7.24	23.99	7.01	96.86	96.07	46.11
IC-3L-3	24.53	7.2	23.89	7.01	97.4	96.06	46.25
IC-3S-1	47.85	7.06	46.74	6.90	97.67	94.73	43.99
IC-3S-2	48.06	7.09	46.96	6.93	97.69	94.82	43.87
IC-3S-3	48.83	7.21	47.57	7.03	97.41	94.42	44.29
IC-48L-1	21.99	6.37	21.03	6.09	95.65	96.09	44.05
IC-48L-2	23.48	6.83	22.58	6.56	96.15	96.42	43.7
IC-48L-3	20.69	6.02	19.93	5.8	96.31	95.85	44.68
IC-48S-1	24.92	7.24	23.96	6.96	96.14	95.38	45.2
IC-48S-2	21.75	6.48	21.55	6.41	99.05	95.61	43.19
IC-48S-3	22.18	6.43	21.26	6.16	95.82	96.84	44.56
PBS-3L-1	24.76	7.19	23.77	6.9	96.02	95.98	44.58
PBS-3L-2	24.4	7.13	23.64	6.91	96.87	96.22	45.63
PBS-3L-3	24.72	7.23	23.97	7.02	96.98	95.78	45.65
PBS-3S-1	25.27	7.34	24.24	7.05	95.94	96.36	44.81
PBS-3S-2	25.1	7.24	23.94	6.9	95.35	96.41	40.65
PBS-3S-3	20.99	6.08	20.09	5.82	95.7	96.22	44.19
PBS-48L-1	23.13	6.76	22.42	6.55	96.92	96.07	44
PBS-48L-2	24.1	7.03	23.3	6.8	96.69	96.01	44.44
PBS-48L-3	22.57	6.53	21.55	6.24	95.51	95.96	43.75
PBS-48S-1	23.94	6.95	22.98	6.68	96.01	96.21	42.74
PBS-48S-2	25.4	7.34	24.16	6.98	95.14	96.93	43.08
PBS-48S-3	24.99	7.26	24.02	6.98	96.13	96.01	43.9

## Data Availability

The data presented in this study are available in this article. The raw sequencing reads from the RNA-seq described in this study have been deposited in the NCBI Sequence Read Archive under accession number [PRJNA1296786] (https://www.ncbi.nlm.nih.gov/bioproject/PRJNA1296786, (accessed on 12 August 2025)).

## Supplementary Materials

The following supporting information can be downloaded at: https://www.mdpi.com/article/10.3390/ani15162454/s1.
